# Level, trends and factors associated with early seeking care for children under five with a fever in Guinea

**DOI:** 10.1016/j.parepi.2025.e00459

**Published:** 2025-09-19

**Authors:** Sidiki Kaba, Mamadou Dian Dilé Diallo, Facinet Conté

**Affiliations:** a Université Paris 1 Panthéon Sorbonne, France; b Direction Nationale Population Développement, Guinea; c Université Général Lansana Conté de Sonfonia-Conakry, Guinea

**Keywords:** Fever, Malaria, Children under 5, Early care, Guinea

## Abstract

Malaria remains the leading cause of morbidity in Guinea. To contain it, the National Malaria Control Programme, following the guidelines of the World Health Organisation, prioritises the mass distribution of insecticide-treated mosquito nets, seasonal chemoprevention, intermittent preventive treatment for pregnant women and the proper management of all confirmed cases. The latest survey on malaria and anaemia indicators conducted in 2021 indicates a malaria prevalence of 17 % among children under five. Among these children who had a fever in the two weeks preceding the survey, only 32 % of cases were treated within 48 h in 2021, compared to 37 % in 2018. This represents a decline in the use of health care services, even though the prevalence of malaria remains high. In light of this observation, it is essential to identify the factors that explain this low use of health care and/or treatment for fever in children under five in Guinea.

In this context, the data used comes from the 2018 Demographic and Health Survey and the 2021 Malaria and Anaemia Indicator Survey. Factors associated with early recourse to care for children with fever in Guinea are identified through descriptive and explanatory analyses.

The results show that early recourse to care for children with fever is low and declining, while the prevalence of malaria infections has remained unchanged (17 %) since 2018. Indeed, early recourse to health care for children is low in rural areas, in poor households and in the regions of Boké, Kindia, Labé and Nzérékoré.

Research into the factors associated with children suffering from fever seeking care early, particularly in endemic areas, could help to identify new strategies for eradicating malaria in Guinea. The impact of such research could be profound, saving countless lives and improving the health of the country's children.

## 1 Background

Malaria remains the leading cause of morbidity and mortality in developing countries, particularly in Africa (Mutombo et al., 2018). In 2022, malaria infections worldwide were estimated at 249 million and caused 608,000 deaths. Nearly 94 % of malaria infections and 95 % of malaria deaths occur in Africa (WHO, 2023). However, in 2015, the international community pledged to reduce malaria incidence and mortality by at least 90 % by 2030 (WHO, 2021). This political commitment is reflected in the national malaria control programme implemented in endemic regions in Africa, where the risk of infection remains persistent. This programme needs to be strengthened to eliminate infections that are a public health problem in the African region, particularly in Guinea.

The early use of care in febrile children under five has decreased over the period of 2018–2021, despite the increase in fever. In fact, the percentage of children receiving early care was 37 % in 2018 compared with 32 % in 2021, while the prevalence of fever cases increased from 17 % to 23 % over the same period (NIS and ICF, 2021; Das and Ravindran, 2010). This trend in the use of health care reflects the disengagement of families in the care of febrile children exposed to malaria infections, despite the diversity of sources of advice and treatment (public hospitals, communal medical centres, health centres, health posts, community health workers, community relays, pharmacies, private doctors, private health care practices, other medical sectors, traditional practitioners, friends and relatives). Early treatment of fever is one of the strategic recommendations in the fight against malaria (Das and Ravindran, 2010; Awantang et al., 2018). The persistently low level of treatment uptake shows that this is an area that needs to be addressed (Deressa, 2007; Birhanu et al., 2016) if we are to reduce the incidence and mortality rates associated with malaria.

The aim of this study is to analyse the factors associated with early recourse to treatment in children under five with fever in the two weeks preceding the survey in Guinea. It will enable the national malaria control programme to achieve its objectives by improving knowledge of the main factors associated with early recourse to treatment for fever in children under five. Treatment is considered early if it is sought within 48 h of the onset of fever, i.e. on the same day or the following day (NIS and ICF, 2021). The use of health care services includes health facilities (public and private), health care providers, pharmacies, traditional practitioners and others.

In terms of explanatory variables, the sex and age of the child play a central role in demographic analysis, as several case studies have highlighted (Franckel, 2004; Faye, 2022). In terms of the characteristics of mothers/caregivers, education improves the couple's income and their ability to pay for health care. It also influences the choice of health care and the perception of the seriousness of illnesses (Baxerres and Le Hesran, 2004). It plays a decisive role in therapeutic practices by acting on the socio-cultural constraints that trivialise the disease (Benyoussef and Wessen, 1974; Caldwell, 1979; Cleland et al., 1989). Uneducated mothers, anxious to protect their children from misfortune, prefer not to talk about their state of health (Kaba, 2020, Kaba, 2024). A mother's level of education encourages her children to be vaccinated (Diallo, 2021) and reduces the risk of mortality in the first 5 years of life (Kaba, 2020; Kaba et al., 2020). A mother's age determines her physical health and reproductive capacity (Hamadou Daouda, 2012). Household characteristics (standard of living quintile, gender of head of household, number of children, area of residence and region of residence) that determine the level of resources are associated with early use of health care (Kane, 2017). Indeed, reviews of the literature show that the use of health care increases as the standard of living improves (Faye, 2022; Manzambi et al., 2000; Saidou, 2018; Ngwen, 2018), that the use of health care is higher among children living in male-headed households than elsewhere (Faye, 2022) and in urban areas than in rural areas (Faye, 2022; Cissé and Sauvain-Dugeedil, 2018).

The authors use data from the 2021 Malaria and Anaemia Indicator Survey (MAIS) and the 2018 Demographic and Health Surveys (DHS) to assess the prevalence and trends in fever care-seeking by child, maternal and household characteristics. Factors are modelled using binary logistic regression, taking into account the complex sample design, to assess the drivers of reluctance to seek early care for children and the trends needed to strengthen malaria control programmes.

## 2 Methods

### 2.1 Data source

The data used in this study come from the 2018 Demographic and Health Survey and the 2021 Malaria and Anaemia Indicator Survey (NIS and ICF, 2021; NIS and ICF, 2018). These two surveys are part of the Demographic and Health Survey programme, supported by 10.13039/100000200USAID and implemented by ICF. They are nationally representative and cover urban and rural areas as well as each administrative region of the country. The information on fever and malaria concerns prevalence, use of care and treatment, and the type of drugs used to treat fever/malaria. These surveys are carried out in Guinea by the National Institute of Statistics in collaboration with the National Institute of Public Health, with technical assistance from the DHS programme during all phases of the surveys. The protocol and data collection tools have been validated by the National Committee for Ethics and Health Research and ICF Review Board.

The results of the 2021 Malaria and Anaemia Indicator Survey and those of the 2018 Demographic and Health Surveys are representative for the country as a whole, by administrative region and by area of residence. Each region has an urban stratum and a rural stratum, with the exception of the Conakry region, which is essentially urban. Then, in each stratum, the sample is constituted using a stratified survey by zone and drawn in 2 stages. At the first stage, enumeration areas are systematically drawn with a probability proportional to the number of households from a list drawn up during the 2014 general population and housing census. At the second stage, a fixed sample of households is systematically drawn with equal probability from each enumeration area. Then, all women aged between 15 and 49, usually living in the selected households or having spent the night before the survey, were eligible for the survey, and all children under 5 were eligible for biological tests and measurements (NIS and ICF, 2021; Das and Ravindran, 2010). Individual data files from these surveys can be downloaded from the DHS Programme website.

### 2.2 Target population

The target population was children under five who had had a fever in the two weeks preceding the survey.

### 2.3 Description of variables

The result variable takes the value 1 if the child was seen within 48 h of the onset of fever, as recommended by the WHO, and 0 otherwise. Sex has two modalities (1: male and 2: female), and the child's age in months is recorded as <24 and 24 +. The mother's level of education (no level, primary, secondary +) and her age (<20, 20–34 and 35+ years) are recorded. The number of children in the household is grouped into three classes (<3, 3–4 and 5+). The household's standard of living (very poor, poor, average, rich and very rich), place of residence (urban and rural) and region (Boké, Conakry, Faranah, Kankan, Kindia, Labe, Mamou and Nzerekore) are unchanged.

### 2.4 Analysis methods

This study combines descriptive and explanatory analyses. Descriptive analysis aims to synthesise and clarify the distribution of explanatory variables between outcome groups, while binary logistic regression identifies and evaluates the influence of explanatory factors, taking into account the complexity of the sampling design.

## 3 Results

### 3.1 Prevalence of seeking care for children under 5 with fever

Nationally, the percentage of children under 5 with fever in the past two weeks before the interviewer's visit for whom care was sought within 48 h of fever onset decreased by 5 percentage points ([per = 37 %, 95 % CI = 33.4–40.1] to [per = 32 %, 95 % CI = 28.1–36.5]) from 2018 to 2021. Early care-seeking for fever among children under 5 varies by child, maternal, and household characteristics (Table 1).

**Table 1:** percentage of children under 5 with fever receiving early treatment.

		Early seeking care in case of fever 2018				Early seeking care in case of fever 2021			
		Percent	95 % CI		Effective	Percent	95 % CI		Effective
Sex of child									
Male		35.5	31.3	39.9	653	31.1	26.6	36.0	494
Female		38.0	33.9	42.3	581	33.4	27.4	39.9	427
Age of the child (month)									
<24		36.0	31.7	40.6	600	34.0	29.0	39.5	397
24+		37.3	33.1	41.6	634	30.7	25.9	35.9	524
Age of mother									
<20		26.7	18.6	36.8	98	28.0	18.2	40.4	76
20–34		35.6	31.7	39.7	827	32.4	28.0	37.1	644
35 +		42.6	35.6	50.0	310	33.0	26.0	40.7	201
Mother's education level/guardian									
No education		35.2	31.4	39.1	932	27.9	23.5	32.7	570
Primary		34.4	27.0	42.6	137	39.2	31.0	48.0	198
Secondary +		46.9	38.6	55.3	166	39.8	31.0	47.4	153
Residential areas									
Urban		48.3	42.4	54.1	401	41.6	34.6	49.1	251
Rural		31.1	27.2	35.3	834	28.6	24.1	33.6	670
Region									
Boké		32.8	24.3	42.5	94	19.3	11.0	31.7	112
Conakry		52.3	42.9	61.6	170	49.8	39.5	60.1	119
Faranah		26.2	17.0	38.2	104	57.0	38.2	74.0	78
Kankan		41.6	34.9	48.7	204	33.2	25.2	42.3	198
Kindia		38.1	31.6	45.0	294	28.1	19.5	38.6	141
Labe		6.5	2.7	14.5	111	22.1	14.7	31.9	95
Mamou		38.2	27.2	50.6	144	35.5	25.1	47.6	47
Nzérékoré		40.8	28.4	54.5	113	20.9	13.2	31.6	131
Household head									
Male		37.0	33.5	40.6	1081	31.6	27.5	36.0	801
Female		34.4	26.7	43.1	153	35.8	26.2	46.7	120
Number of children in the household									
<3		38.0	34.0	42.1	865	29.9	25.7	34.5	580
3–4		34.5	28.8	40.7	298	35.7	27.7	44.5	273
5+		29.5	19.1	42.6	72	36.9	23.2	53.2	68
Household well-being quintile									
Poorest		20.1	15.4	25.8	258	21.8	14.8	30.9	200
Poorer		33.3	26.6	40.6	259	25.9	20.0	33.0	202
Middle		33.8	27.4	40.7	244	34.7	27.5	42.7	199
Richer		46.5	38.6	54.6	235	37.2	27.3	48.2	174
Richest		51.5	44.4	58.4	239	45.2	36.6	54.1	147
**Total**		**36.7**	**33.4**	**40.1**	**1234**	**32.1**	**28.1**	**36.5**	**921**

Indeed, between 2018 and 2021, early recourse to care in case of fever decreased from 36 % (95 % CI = 31.3–39.9) to 31 % (95 % CI = 26.6–36.0) in boys and in girls from 38 % (95 % CI = 33.9–42.3) to 33 % (95 % CI = 27.4–39.9). According to age, low use of care in case of fever is observed in children 24 months and older ([per = 31 %, 95 % CI = 25.9–35.9, age ≥24 + months], [per = 34.0, 95 % CI = 29.0–39.5 age < 24 months]) in 2021, whereas in 2018 ([per = 37 %, 95 % CI = 33.1–41.6], [per = 36 %, 95 % CI = 31.7–40.6]), there was no disparity between age groups (Table 1).

The percentage of children receiving health care increased with maternal age and education level in 2018 and 2021. Among mothers aged younger than 20 years to those aged 35 years or older, the percentage of children receiving health care increased from 27 % (95 % CI = 18.6–36.8) to 43 % (95 % CI = 35.6–50.0) in 2018, but it did not increase significantly in 2021 (28 % [95 % CI = 18.2–40.4] to 33 % [95 % CI = 26.0–40.7]). In 2018, early care use was lower for children born to adolescent mothers and higher for children born to mothers aged 35 years or older. However, this gap narrowed in 2021 (Table 1).

The same trend is observed for the level of education, with 35 % (95 % CI = 31.4–39.1) of children born to mothers with no education, 47 % (95 % CI = 38.6–55.3) of those born to mothers with a secondary education or higher in 2018 and 28 % (95 % CI = 23.5–32.7) and 40 % (95 % CI = 31.0–47.4) respectively in 2021. According to the level of education, the percentage increased in 2018 and in 2021 (Table 1).

The percentage of children with fever seeking care within 48 h of symptom onset varies by income quintile. From the lowest to the highest quintile, the percentage increases from 20 % (95 % CI = 15.4–25.18) to 52 % (95 % CI = 44.4–58.4) in 2018 and from 22 % (95 % CI = 14.8–30.9) to 45 % (95 % CI = 36.6–54.1) in 2021. The relationship between the number of children in the household and the use of their health care is ambiguous. Indeed, the increase in the number of children is accompanied by a reduction in the use of their health care in 2018, while the opposite effect occurs in 2021. Indeed, when the number of children in the household increases from less than 3 to 5 or more, the 2018 statistics show a decrease in the use of care from 38 % (95 % CI = 34.0–42.1) to 30 % (95 % CI = 19.1–42.6) while the 2021 statistics indicate an increase from 30 % (95 % CI = 25.7–34.5) to 37 % (95 % CI = 23.2–53.2). A similar contradiction emerges according to the sex of the head of household. The percentage of seeking care for children with fever is higher among male heads of household (pr = 37 %, 95 %CI = 33.5–40.6) in 2018 than among female heads of household (pr = 34 %, 95 %CI = 26.7–43.1), while in 2021, the percentage is higher among female heads of household (pr = 36 %, 95 %CI = 26.2–46.7) than among male heads of household (pr = 32 %, 95 %CI = 27.5–36.0). Finally, the standard of living of the household increases the use of care, but the number of children as well as gender do not determine the direction of its variation (Table 1).

Early access to care for children with a fever is low in rural areas. The percentage of rural children with fever seeking care early was 31 % (95 % CI = 27.2–35.3) in 2018 and 29 % (95 % CI = 24.1–33.6) in 2021, while the percentage of urban children was 48 % (95 % CI = 42.4–54.1) in 2018 and 42 % (95 % CI = 34.6–49.1) in 2021. Low referrals were recorded in the Boké region in 2018 (pr = 33 %, 95 % CI = 24.3–42.5) and 2021 (pr = 19 %, 95 % CI = 11.0–31.7), and in the Labé region in 2018 (pr = 7 %, 95 % CI = 2.7–14.5) and 2021 (pr = 22 %, 95 % CI = 14.7–31.9) compared to other regions. These results indicate that early recourse to health care for children with fever is low in the Boké and Labé regions, and in rural areas (Table 1).

### 3.2 Binary logistic regression in complex sampling design

The likelihood of early care seeking among children with a fever did not vary significantly by their characteristics. Girls and boys with fever had similar probabilities, and age had no effect on early care seeking (Table 2).

**Table 2:** Odds ratios of factors associated with early care seeking in children under 5 with fever.

	Adjusted model, DHS 2018				Adjusted model, MIS 2021			
Variable	aOR	95 %CI		*P*-Value	aOR	95 % CI		P-Value
Sex (ref: male)								
Female	1.14^ns^	0.90	1.45	0.2720	1.18^ns^	0.85	1.65	0.3211
Age of the child (ref: <24 months)								
24+	0.95^ns^	0.73	1.23	0.6996	0.85^ns^	0.63	1.14	0.2701
Age of mother (ref: <20 years)								
20–34	1.52^ns^	0.91	2.54	0.1088	1.22^ns^	0.68	2.18	0.5053
35 +	2.28**	1.27	4.11	0.0062	1.43^ns^	0.73	2.82	0.2959
Education level of mother/guardian (ref: no education)								
Primary	0.82^ns^	0.55	1.21	0.3126	1.60**	1.03	2.50	0.0365
Secondary+	1.00	0.64	1.59	0.9832	1.31^ns^	0.77	2.25	0.3140
Household head (ref: male)								
Female	1.08^ns^	0.70	1.68	0.7281	1.22^ns^	0.73	2.04	0.4442
Number of children (ref: <3)								
3–4	0.95^ns^	0.70	1.31	0.7744	1.18^ns^	0.79	1.77	0.4127
5+	0.56**	0.31	0.99	0.0475	1.40^ns^	0.74	2.65	0.2975
Place of residence (ref: rural)								
urban	0.90^ns^	0.55	1.45	0.6581	0.85	0.41	1.75	0.6614
Region (ref: Boké)								
Conakry	1.36^ns^	0.73	2.54	0.3386	2.91**	1.22	6.93	0.0164
Faranah	0.80^ns^	0.40	1.60	0.5194	6.16***	2.28	16.65	0.0004
Kankan	1.28^ns^	0.75	2.18	0.3608	2.11^ns^	0.86	5.20	0.1045
Kindia	1.18^ns^	0.71	1.94	0.5223	1.54^ns^	0.63	3.75	0.3404
Labe	0.16***	0.06	0.42	0.0002	1.19^ns^	0.45	3.12	0.7218
Mamou	1.25^ns^	0.66	2.35	0.4941	2.21*	0.86	5.66	0.0986
Nzérékoré	1.36^ns^	0.67	2.75	0.3920	1.96^ns^	0.50	3.20	0.6174
Household well-being quintile (ref: Poorest)								
Poorer	1.80**	1.18	2.76	0.0067	1.39^ns^	0.80	2.40	0.2362
Middle	1.88**	1.25	2.83	0.0027	1.97**	1.07	3.63	0.0294
Richer	3.35***	2.02	5.56	0.0000	1.95*	0.89	4.29	0.0951
Richest	3.84***	2.04	7.22	0.0000	2.40*	0.96	5.99	0.0615
_cons	0.16***	0.08	0.34	0.0000	0.10***	0.03	0.30	0.0001
Effective	1221				921			

ns *p* < 1, ^⁎^*p* < 0.10, ^⁎⁎^*p* < 0,05, ^⁎⁎⁎^*p* < 0.01.

On the other hand, maternal factors influence early care seeking. The likelihood of early care seeking among children with fever is higher among mothers aged 35 and over (aOR = 2.28, 95 % CI = 1.27–4.11) than among adolescent mothers (under 20 years) in 2018, but the difference is not significant in 2021. As for the mother's education level, no significant difference was observed in the 2018 data. However, in 2021, children with a fever of mothers with a primary education level have a higher probability of early care seeking (aOR = 1.60, 95 % CI = 1.03–2.50) than their counterparts of mothers with no education level (Table 2).

The analysis also shows that household characteristics influence early seeking care for fever. Feverish children are more likely to benefit from early care in middle well-being (aOR = 1.88, 95 % CI = 1.25–2.83), richer households (aOR = 3.35, 95 % CI = 2.02–5.56) and richest households (aOR = 3.84, 95 % CI = 2.04–7.22) than poorest, and the results from 2021 are consistent with those from 2018. However, the influence of the number of children under 5 in the household on the use of care is not significant, except in 2018, where feverish children are less likely to benefit from early consultation when their number increases in the household (number = 5+, aOR = 0.56, 95 % CI = 0.31–0.99). Regarding the gender issue, feverish children have the same probability of seeking care among female heads of household as among male heads of household (Table 2).

The effects of residential areas are not significant in 2018 and 2021. Furthermore, in some regions, children are more likely to receive health care than in others. These include the Faranah region (aOR = 6.2, 95 % CI = 2.28–16.65) and Conakry (aOR = 2.9, 95 % CI = 1.22–6.93) in 2021, but the other regions are no different from Boké (Table 2).

## 4 Discussions

Sex and age are central variables par excellence in demographic analysis. Various studies have examined their associations with the use of health care in children (Franckel, 2004; Faye, 2022). Seeking care is the same for girls as for boys, regardless of age. These differences are thought to result from the fact that malaria awareness messages in the media do not differentiate the risk by sex or age of the child. The recent 2021 Malaria and Anaemia Indicator Survey showed that more than half of women (58 %) saw or heard a message about malaria in the last six months. These messages often concern the risk, severity, and consequences of late care-seeking, particularly among children (regardless of sex and age) and pregnant women, who are the most vulnerable group. Then, many women fear the onset of fever as a sign of malaria infection, regardless of sex and age. Therefore, treatment of fever is necessary for both girls and boys, regardless of age (NIS and ICF, 2021). Other studies have also shown that gender does not determine health-seeking behaviour in sick children (Franckel, 2004; Faye, 2022; Nganda Mekogo, 2012). Similarly, health-seeking recommendations apply to all children under 5 years of age (WHO, 2008).

Various studies have also examined the influence of maternal age on children's access to care in case of illness. It appears that the choice of care depends strongly on the perception of the severity of the disease (Baxerres and Le Hesran, 2004). The results of the 2021 Malaria and Anaemia Indicator Survey showed that mothers have relatively the same level of information. Exposure to malaria messages in the media, perception of risk and severity are relatively invariant according to age (NIS and ICF, 2021). This explains the non-association between maternal age and the need for care in children in case of fever. Other studies have also reached the same conclusion (Nganda Mekogo, 2012).

Education plays an important role in children's health. It improves the couple's income and health care expenses. It facilitates access to messages about malaria and influences behaviours that trivialise diseases. Concerned about protecting their children from bad luck, poorly educated women often prefer to keep their health status secret (Nganda Mekogo, 2012), sometimes trivialising childhood illnesses. However, thanks to the mother's education, such beliefs are eradicated. In health, the role of education is widely documented in vaccination (Diallo, 2021) and mortality of children under 5 (Kaba, 2024). Indeed, this study shows that improving the level of education increases the chances of children with fever receiving care. Then, the percentage of children for whom care is sought within 48 h of fever increases when the mother's education level improves. Similar results have been found elsewhere (Nganda Mekogo, 2012; Yaogo et al., 2014; Dieng et al., 2014).

Children's health care expenses are mainly borne by the father and mother. The responsibility for this care falls on the father, who rarely seeks help from those around him to avoid exposing himself to shame. The same is true for the mother. Therefore, the level of resources is important for early care seeking (Kane, 2017). The results of this study confirm this, as early care seeking improves with the household's standard of living. Various studies have also reached the same conclusion (Faye, 2022; Manzambi et al., 2000; Saidou, 2018; Ngwen, 2018).

The results of this study showed that the direction of the relationship between the number of children in the household and the use of care for fever is ambiguous. In 2018, children were less likely to receive health care when they were in households with more than 4 children. However, relationships disappeared in 2021. Descriptive statistics also showed that the percentages of care seeking are relatively invariant with the number of children in the household. On the one hand, such a result would mean that the care of children in case of fever is mainly provided by the mother, who rarely seeks help from the head of household unless the latter is the child's father. On the other hand, expenses related to treatment in case of fever could be less expensive. The second hypothesis is the most plausible, since among the priorities of the national malaria control policy are the mobilisation of sustainable financing to fight malaria and the reduction of the malaria burden for the long-term achievement of the goal of eliminating the disease (DNPSC, 2014). This effective involvement of the State facilitates the management of children's health in the process of eradicating malaria in Guinea.

This study found no correlation between male-headed or female-headed households and access to care for children with fever. The percentage of children with fever receiving care was independent of whether the household was headed by a woman or a man. This finding is believed to be related to the efforts of the malaria control programme, not only to raise public awareness but also to facilitate universal access to health care. However, other studies have shown greater dependency among children when the household is headed by a man rather than a woman (Faye, 2022).

Environment and region of residence are important variables in spatial analyses. They provide information on the impact of health programmes and behavioural differences in different intervention areas. Malaria control programmes are often implemented based on the endemic area. Thus, they differ from one region to another and from rural to urban areas. Furthermore, the results of this study show that health care utilisation is higher in urban areas than in rural areas. This reflects the lack of qualified health personnel, insufficient health services, language barriers to malaria messages in the media, precarious living conditions, and the influence of sociocultural constraints in rural areas. Studies have also shown that inequalities in access to health care are more pronounced in rural areas than in urban areas (Faye, 2022; Cissé and Sauvain-Dugeedil, 2018).

## 5 Conclusion

The results of this study highlighted factors associated with early care seeking among children with fever. These include the mother's education level, household standard of living, and the area and region of residence. Conversely, care seeking among children with a fever does not differentiate according to the child's sex and age. There is no difference between households headed by men and those headed by women. In terms of policy implications, the national malaria control programme would benefit from directing its interventions more toward rural areas, which have so far been disadvantaged, despite national efforts. Given the commitment to eliminate malaria in Guinea and the current level of early care seeking among children with fever, it is important to further strengthen current strategies in the eight regions, with a greater emphasis on the regions of Boké, Kindia, Labé, and Nzérékoré, where early care seeking is lowest.

The results of this study highlight both strengths and limitations. Its relevance is twofold: from a political perspective, it is part of the objective of significantly reducing malaria transmission within the population with a view to its elimination, as emphasised by the National Malaria Control Programme; from a scientific perspective, several studies have not taken into account the hierarchical data structure advocated by the DHS programme. In terms of limitations, the lack of stratification by malaria-endemic areas masks common and specific factors related to the early management of fever in children under 5.

In terms of perspective, the previous paragraph highlights the need to analyse regional inequalities in the early use of care in children with fever to identify common and specific factors and share knowledge and experiences in order to better plan interventions in regions highly exposed to malaria infection in Guinea.

## CRediT authorship contribution statement

**Sidiki Kaba:** Writing – review & editing, Writing – original draft, Visualization, Methodology, Formal analysis, Conceptualization. **Mamadou Dian Dilé Diallo:** Writing – review & editing, Writing – original draft, Visualization, Supervision, Formal analysis, Data curation, Conceptualization. **Facinet Conté:** Writing – original draft, Supervision, Methodology.

## Funding

There was no funding.

## Declaration of competing interest

Not applicable for that section.

## Data Availability

Data will be made available on request.
